# Myocardial function in patients with anomalous left coronary artery from the pulmonary artery syndrome: A long-term speckle tracking echocardiographic study

**DOI:** 10.1371/journal.pone.0223227

**Published:** 2019-10-15

**Authors:** Alicja Dąbrowska-Kugacka, Karolina Dorniak, Jarosław Meyer-Szary, Agnieszka Herrador Rey, Ewa Lewicka, Katarzyna Ostrowska, Joanna Kwiatkowska

**Affiliations:** 1 Department of Cardiology and Electrotherapy, Medical University of Gdansk, Gdansk, Poland; 2 Department of Noninvasive Cardiac Diagnostics, Medical University of Gdansk, Gdansk, Poland; 3 Department of Pediatric Cardiology and Congenital Heart Disease, Medical University of Gdansk, Gdansk, Poland; 4 Department of Cardiology, Polish Mother’s Memorial Hospital–Research Institute, Lodz, Poland; Universita degli Studi di Roma La Sapienza, ITALY

## Abstract

**Background:**

Anomalous origin of the left coronary artery (LCA) from the pulmonary artery (ALCAPA) is a rare congenital heart disease. Retrograde flow from the right coronary artery (RCA) through natural collaterals to the low-pressure main pulmonary artery causes extensive ischemia. Limited data concerning the extent of permanent myocardial damage and functional recovery after surgical repair in the long-term follow-up is available.

**Aim:**

Determination of the incidence of incipient myocardial dysfunction in ALCAPA patients in the long-term observation using tissue Doppler and speckle tracking echocardiography.

**Methods and results:**

Eighteen ALCAPA patients after surgical repair (at median age of 7 months, range 3–167) underwent echocardiographic examination after (median) 17 years. All but 4 patients in NYHA class II presented well at follow-up. No narrowing in proximal LCA was detected in color Doppler. The initial (pre-surgical) left ventricular (LV) ejection fraction of 33±17% almost normalized to 55±6%, but was lower than in the age, sex and body surface area matched control group: 62±5% (p<0.001). At follow-up, LV global longitudinal strain (LS): -15.8±3.3% vs -21.9±1.7%; right ventricular LS: -20.6±3.9% vs -24.9±4.6%; left atrial LS: 27.7±4.3% vs 41.0±11.5%; right atrial LS: 26.8±7.4% vs 44.0±7.9% and early pulsed wave to tissue Doppler mitral filling ratio (E/E’): 8.1±2.6 vs 5.8±1.3 were impaired in the ALCAPA population in comparison to the control group (p<0.01 for all comparisons). LV radial and circumferential strain did not differ between groups. Mean LS in the ALCAPA patients in the RCA region was -19.0±4.4%, while in the LCA region -13.8±7.3% (p<0.00001).

**Conclusions:**

Despite good clinical condition and normalized LV ejection fraction in ALCAPA patients after surgical repair in the long-term follow-up, the diastolic and longitudinal systolic function of all cardiac chambers remained impaired, especially in the LCA region. Lifelong surveillance of repaired ALCAPA patients is needed.

## Introduction

Anomalous origin of the left coronary artery (LCA) from the pulmonary artery (ALCAPA), also known as Bland-White-Garland syndrome, is a rare congenital heart disease, occurring approximately in 1 per 300.000 live births [1]. Retrograde flow in the LCA supplied from the right coronary artery (RCA) through natural collaterals to the low-pressure main pulmonary artery causes extensive myocardial ischemia. Ischemia occurs due to loss of perfusion pressure in the coronary arteries resulting from this specific steal syndrome. Most often it manifests in early childhood with symptoms of congestive heart failure or myocardial ischemia, and if left untreated the mortality reaches 90% in the first year of life. Preoperative myocardial ischemia results in reduced left ventricular (LV) ejection fraction (LVEF), mitral regurgitation, and LV dilation [2]. The treatment of choice is immediate surgical reimplantation of the LCA into the aorta [3] or creation of intrapulmonary baffle when coronary translocation is not feasible (Takeuchi repair) [4].

Due to the rareness of the condition limited data regarding long-term follow-up is available but most reports indicate that early diagnosis and immediate surgical intervention leads to excellent results, with normalization of LV size and function, resolution of mitral regurgitation, especially when operated in early childhood [5–7]. However, other studies report on persistent mitral regurgitation, arrhythmia, sudden cardiac death, exacerbation of heart failure or coronary stenosis, necessitating surgical reintervention or even heart transplantation [2,8,9]. Some authors underline the persistence of chronic myocardial injury, including wall motion abnormalities, perfusion defects, and scarring [10, 11, 12]. Most studies, however, confine to cardiac chambers dimensions and LVEF assessment [13]. As myocardial ischemia and fibrosis appear to deteriorate the clinical course after corrective surgical intervention [14], detection of even minor abnormalities in the ALCAPA population is of paramount importance. There was one late arrhythmic death due to ventricular fibrillation in an asymptomatic patient with normal LV function at the age of 21. This fact motivated us to invite all our ALCAPA patients for a detailed follow-up examination and to summarize these observations [15].

In the last years, myocardial strain imaging has become more frequently used for the assessment and quantification of regional ventricular abnormalities. This very sensitive method enables the evaluation of myocardial function, well beyond standard echocardiographic techniques, and detection of early subclinical myocardial abnormalities, even in the presence of normal LVEF [16].

The aim of this study was to determine the incidence of myocardial dysfunction by means of tissue Doppler and speckle tracking echocardiography (STE) in the long-term observation of the ALCAPA patients operated in their childhood.

## Materials and methods

Clinical data of all patients with ALCAPA under observation in our Clinical Hospital between 1991 and 2014 (24 cases) are presented as a short communication in a separate publication [15]. Four patients of this study population died and 2 did not have detailed echocardiographic examination performed. The study population presented in the current paper consisted of 18 ALCAPA patients (12 females), operated at the median age of 7 months (range 3–167 months). Clinical and imaging data at diagnosis, at the time of the surgical procedure and one year after the operation were obtained from patients’ documentation. The clinical status of the patients was evaluated according to the American Hospital Association [17]. The follow-up examination was performed after the median of 17 years (range 4–28 years) from surgery. At the final follow-up visit all patients underwent physical and echocardiographic examination. Taking into consideration the diverse age of the study population (pediatric, adolescents and adults) and the difficulty of extrapolating the echocardiographic values to the z-score, an age, sex and body surface area matched healthy control group (n = 18) was included in our study.

The study protocol was approved by the local ethics committee (Medical University of Gdansk, Poland; NKBBN/278/2017) and the consent for the use of anonymised echocardiographic and clinical data for research purposes was obtained from all patients, their parents or legal guardians.

### Echocardiographic examination

The echocardiographic data at diagnosis and one year after operation were gathered from the medical documentation. The LVEF in these two examinations was calculated on the basis of the Teichholz formula, with LV end-diastolic (LVEDd) and end-systolic (LVESd) diameters measured in the parasternal long-axis view.

The final follow-up examination was performed according to the guidelines [18] at rest in left lateral decubitus position without sedation, using a commercially available system (Vivid E9, GE Healthcare, Horten, Norway) and a 3.5-MHz phased transducer. One physician with 25 years of experience in echocardiography (ADK) performed all recordings and measurements. Images from 3 cardiac cycles triggered by the R wave of the QRS complex were digitally saved in cine loop format. All images were stored for further off-line analysis using software for echocardiographic quantification (EchoPac 201, GE Healthcare, Norway). The following parameters were measured in the parasternal long-axis view: LVEDd, LVESd, LV wall thickness, right ventricular (RV) outflow tract diameter and antero-posterior left atrial (LA) diameter. Taking into consideration the pediatric age range the z-score for LVEDd and LVESd was also calculated [19]. The LV end-systolic and end-diastolic volumes were obtained from the apical 2- and 4-chamber views, and LVEF was calculated by the Simpson’s biplane method. Left and right atrial (RA) areas were calculated in end-systole from the 4-chamber apical view. Systolic aortic and pulmonary, as well as tricuspid regurgitant jet maximal velocities were obtained by the continuous wave Doppler from the appropriate views. Special attention was focused on the degree of mitral regurgitation and the presence of fibroelastosis of the papillary muscles. Whenever possible, the flow in the proximal segments of LCA and RCA were assessed by color Doppler.

Additionally, in order to evaluate the longitudinal RV and LV function, peak systolic velocities in the basal segments of lateral LV wall (S‘LW), interventricular septum (S‘IVS) and lateral RV wall (S’RV) by spectral tissue Doppler, obtained from the apical 4-chamber view were measured. The RV systolic function was also evaluated based on the tricuspid annular plane systolic excursion (TAPSE) measurement.

The LV diastolic function was evaluated according to the guidelines [20]. The following parameters were measured: early (E) and atrial (A) wave velocity of the mitral inflow assessed by pulsed-wave Doppler at the level of the mitral valve, peak early diastolic velocities (E‘) by spectral tissue Doppler (mean value from the lateral LV wall and IVS), the E/E‘ ratio, and the right lower pulmonary vein inflow pattern obtained from the 4-chamber apical view by pulsed-wave Doppler.

Myocardial strain was measured in all cardiac chambers. The degree of deformation is reported as percentage of peak longitudinal strain (LS) in systole. Decreased myocardial shortening (impaired function) is represented by lower absolute values. Normal values vary by age, and in the study by Marcus et al., which provided reference values of LV strain in the pediatric population, LS values were the lowest in the youngest and oldest age groups [21]. That is why a detailed echocardiographic examination was also performed in the age, sex and body surface area matched healthy control group. The normal value of the LV global LS (LV_GLS) is most often defined as < -18.9%, however other studies report on the value of < -15.9%, as abnormal and this value was considered as the borderline in our study [22]. Aortic valve closure time was determined manually from the pulsed-wave Doppler tracing in the LV outflow tract and used during LS estimation. After initial tracing of the endocardial border and software processing, the operator confirmed adequate tissue tracking. Segments unable to be adequately tracked were excluded.

**Fig 1:**
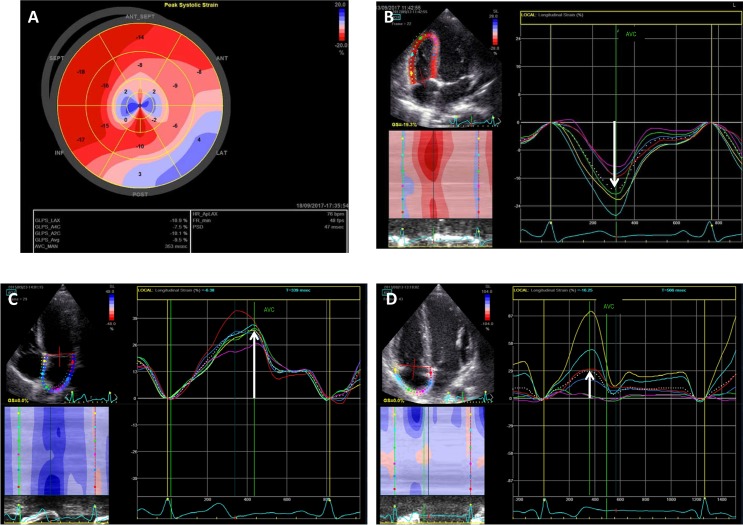
Follow-up 2-dimensional strain evaluation in an ALCAPA patient. (A) Impaired left ventricular global longitudinal strain (LV_GLS = -9,5%) in a patient with akinetic apical and basal postero-lateral segments (blue color); peak longitudinal strain is also calculated separately in each segment; the peak longitudinal systolic strain dispersion (PSD = 47 msec) is calculated automatically (B) right ventricular strain (-19%) (C) left atrial strain (33%) (D) right atrial strain (29%).

Depth-adjusted two-dimensional (2D) LV images were acquired from the apical 2-, 3- and 4-chamber views for off-line measurements of LV_GLS using STE. Frame rates were optimized to > 45 frames/s at the time of acquisition. It was verified, whether peak systolic strain from each LV segment was measured prior to aortic valve closure. The LV_GLS was calculated as the average of peak strain values from 17 LV segments, and LS was also calculated in each segment separately (Fig 1A). The LV was divided into 2 regions: the LCA and RCA region according to the guidelines [18] and the mean LS of each region was calculated. Additionally, the RV strain including the RV free wall and septum (Fig 1B), the LA (Fig 1C) and the RA strain (Fig 1D) were calculated.

In order to determine cardiac contraction synchrony, three parameters were evaluated: 1) atrio-ventricular synchrony, determined as the ratio of diastolic filling period at the level of the mitral valve divided by the RR interval, 2) interventricular synchrony defined as the difference of LV and RV pre-ejection periods obtained by the pulsed-wave Doppler from the respective outflow tracts, and 3) LV intraventricular synchrony, defined as the peak LS strain dispersion (PSD) that was calculated as the standard deviation of the time to peak LS in all LV segments. This parameter was obtained from the automatic LV_GLS analysis.(Fig 1A)

### Statistical analysis

Distribution of the data was assessed by the Shapiro-Wilk test. Data are presented as mean ± standard deviation, median and range (in case of continuous data without normal distribution) or number and percentage of patients. The Student’s *t* test for independent samples was used for normally distributed continuous variables, and the Mann-Whitney U test was applied if variables did not follow a normal distribution. Differences between categorical variables were evaluated using the chi-square test. Echocardiographic parameters were compared between the ALCAPA patients and the control group. P<0.05 was considered statistically significant. All statistical analyses were performed using Statistica 13.1 (PL, Quest Software).

## Results

Clinical data of the study population at the time of ALCAPA diagnosis is presented in S1 Table. In brief, the median age at diagnosis was 6 months (range from 3 to 156 months), and the main reason for hospital admission was enlarged heart silhouette on the chest X-ray performed due to infection. The general condition was good in 8 (44%) and severe in 7 (39%) patients, among whom 2 were in cardiogenic shock. Associated cardiac defects were present in 4 patients: one ventricular septal defect, one type II atrial septal defect, one patent ductus arteriosus and one mild aortic stenosis. The mean LVEF before cardiac surgery was depressed to 33 ± 17% (range 14–69%). The vast majority (80%) of patients showed at least moderate mitral insufficiency and every patient had fibroelastosis of the papillary muscles. There were 15 patients with “infant type” ALCAPA operated (median) 1 month after diagnosis (range 0–2 months) and 3 patients with “adult type” ALCAPA with surgery performed at the age of 8.3, 10.7 and 13.9 years, respectively. All of them were in stable clinical condition at the time of recognition and were operated (median) 10 months after diagnosis (range 10–11 months). Direct reimplantation of the LCA into the aortic root was performed in 14 patients, and 4 patients underwent the Takeuchi repair. None of the patients had mitral valve repair or replacement. After surgery complications occurred in 67% of patients, mainly heart failure exacerbation, however, 1 year after the operation the LVEF was 65 ± 11% and only 1 patient showed LVEF below the lower normal limit (< 55%), namely 30%.

### Long-term follow-up

The follow-up examination was performed median 17 years (range 1.2–23.3) after surgery at the mean age of 16.8 ± 7.8 years. (Table 1) All patients were in very good clinical condition except for 4, who presented heart failure symptoms of NYHA class II– 3 of them were after Takeuchi repair. None of the patients reported chest pain on exercise.

**Table 1:** Clinical data and valvular function of the study population at last follow-up (n = 18).

	mean (± standard deviation) / median (range) or number of patients—n(percentage)
Follow-up [years]	14.5 ± 7.3 / 17.0 (1.2–23.3)
Age at the follow-up examination [years]	16.8 ± 7.8 / 18.8 (3.3–31.3)
Weight [kg]	51 ± 21 / 56 (13–80)
Height [m]	1.54 ± 0.26 / 1.60 (0.99–1.86)
Symptoms of heart failure (NYHA II)	4 (22%)
Systolic murmur	7 (39%)
Valvular function (echo examination):
Aortic stenosis / peak gradient [mmHg]	1 (4%) / 22
Pulmonary stenosis / peak gradient [mmHg]	6 (33%) / 30.6 ± 7.8
Pulmonary insufficiency: mild / moderate	6 (33%) / 3 (17%)
Tricuspid insufficiency	10 (56%)
Mitral insufficiency:	
mild	11 (61%)
moderate	2 (11%)
Fibroelastosis of the papillary muscles	18 (100%)

NYHA–New York Heart Association

In the final follow-up examination the mean LVEF was 55 ± 5.9%. Standard 2-dimensional echocardiography revealed regional LV contraction abnormalities in 6 patients. Hypokinesia was present in the septum, distal segments of the anterior, lateral and posterior walls, while akinesia was limited to the apex and distal segment of the inferior wall. Fibroelastosis of the papillary muscles was present in all patients, however mitral insufficiency improved significantly and only 2 patients (11%) showed moderate and 11 (61%) mild regurgitation. Mild pulmonary stenosis was present in 6 patients with the peak gradient of 30.6 ± 7.8 mmHg, and more than mild pulmonary insufficiency was found in 3 other patients. One patient had mild aortic stenosis (peak gradient of 22 mmHg) present already before ALCAPA operation. Color Doppler echocardiography confirmed patency of the proximal LCA in all patients.

Comparison of the echocardiographic parameters between the ALCAPA patients and the healthy control group is shown in Table 2. Most parameters concerning ventricular and atrial size did not differ between the groups, and only LV end-systolic volume and LV posterior wall diameter were greater in the ALCAPA group. Mean LVEDD z-score at the time of diagnosis in the ALCAPA group was +7.24 (n = 14) and it normalized at follow-up (LVEDD z-score -0.80). The LVEF was significantly lower in the ALCAPA patients, but it was still within normal range. Differences were observed in parameters determining longitudinal function of the LV (S’LW, S’IVS) and the RV (S’RV and TAPSE), which were significantly lower in the ALCAPA patients. Also the intraventricular dyssynchrony (PSD) was more pronounced in this group.

**Table 2:** Comparison of the demographic and echocardiographic parameters between the study population at last follow-up and the control group.

	ALCAPA (n = 18)	Control group (n = 18)	p*
Male (n /%)	6 / 33%	5 / 28%	ns
Age (years)	16.6 ± 8.5	19.7 ± 8.5	*0*.*18*
BSA [m^2^]	1.46 ± 0.43	1.53 ± 0.43	*0*.*72*
HR [bpm]	71 ± 13	75 ± 11	0.32
LVEDd [mm]	45.4 ± 7.8	43.3 ± 6.0	0.37
LVEDd [z-score]	-0.80 ± 0.89	-0.15 ± 1.29	0.09
LVESd [mm]	29.9 ± 7.1	27.0 ± 3.7	0.14
LVESd [z-score]	-0.55 ± 1.07	0.24 ± 1.62	0.09
IVSd [mm]	8.3 ± 1.6	7.4 ± 1.6	0.11
**PWd [mm]**	**8.7 ± 1.4**	**7.3 ± 1.4**	**0.01**
LA [mm]	30.6 ± 7.0	28.3 ± 6.2	0.30
RV [mm]	21.7 ± 4.7	22.0 ± 5.5	0.87
**TAPSE [mm]**	**14.5 ± 3.0**	**21.0 ± 3.3**	**<0.00001**
LVEDvol [ml]	86.0 ± 36.6	69.8 ± 23.6	0.12
**LVESvol [ml]**	**39.0 ± 17.6**	**27.0 ± 10.1**	**0.02**
**LVEF [%]**	**55.0 ± 5.9**	**61.6 ± 4.7**	**<0.001**
RA_area_ [cm^2^]	10.8 ± 2.8	11.1 ± 3.1	0.80
LA_area_ [cm^2^]	14.4 ± 3.8	13.7 ± 4.7	0.61
Ao [mm]	25.3 ± 4.5	23.1 ± 3.9	0.13
MPA [mm]	19.6 ± 3.2	19.7 ± 4.1	0.98
Vmax Ao [m/s]	1.35 ± 0.34	1.28 ± 0.21	0.46
Vmax Pulm [m/s]	1.66 ± 0.85	0.95 ± 0.22	*0*.*06*
TR Vmax [m/s]	2.37 ± 0.39	2.13 ± 0.20	*0*.*29*
**S‘ LW** [cm/s]	**6.9 ± 1.2**	**11.2 ± 2.5**	**<0.00001**
**S‘ IVS** [cm/s]	**6.2 ± 1.3**	**8.6 ± 1.5**	**<0.00001**
**S‘ RV** [cm/s]	**9.3 ± 1.6**	**14.6 ± 1.6**	**<0.00001**
IVMD [ms]	14 ± 15	8 ± 13	0.15
DFP/RR [%]	55 ± 7	54 ± 5	0.81
**PSD [ms]**	**36 ± 11**	**27 ± 6**	***<0*.*01***

n–number, BSA—body surface area, HR–heart rate, bpm–beat per minute, LVEDd–left ventricular (LV) end-diastolic diameter, LVESd–LV end-systolic diameter, IVSd–interventricular septum diameter, PWd–posterior wall diameter, LA–left atrium, RV–right ventricle, TAPSE–tricuspid annular plane systolic excursion, LVEDvol—LV end-diastolic volume, LVESvol—LV end-systolic volume, LVEF–LV ejection fraction, Ao–aortic diameter, MPA–main pulmonary artery diameter, Vmax Ao / Vmax Pulm–maximal velocity through the aortic / pulmonary valve, TR Vmax–maximal velocity of tricuspid regurgitation, S’ LW / S’ IVS / S’ RV–spectral tissue Doppler peak systolic velocity at the basal segment of the lateral wall/ interventricular septum / right ventricle, IVMD–interventricular mechanical delay (difference between left and right ventricular pre-ejection periods), DFP/RR–ratio of the diastolic filling period and RR interval, PSD–peak strain dyspersion

* Student’s t test for independent samples or Mann Whitney U test (italics)

Before the surgery there were 3 patients with LVEF < 20%. All of them showed the „infant type”ALCAPA and one presented with cardiogenic shock at recognition. At the last follow-up after a median of 16.6 years all these patients were asymptomatic. The recovery of the one in cardiogenic shock at recognition was incomplete (LVEF 53%, GLS_LV -13%), the two other recovered very well (LVEF ≥ 55%). No correlation was found between LVEF at recognition and LV function at follow-up. However, after division of the whole ALCAPA group according to LVEF before surgery (< 40% and ≥ 40%) worse RV function was found at follow-up in the group with lower LVEF at recognition (S‘ RV 8.7 ± 1.9 cm/s vs 10.7 ± 1.4 cm/s; p < 0.05).

Parameters of LV diastolic function were worse in the ALCAPA patients than in the control group. (Table 3) Higher mitral A-wave, lower tissue Doppler E’-wave, higher E/E’ ratio, lower systolic and higher diastolic pulmonary vein velocities, lower systolic to diastolic pulmonary velocity ratio, and higher reverse atrial wave velocity were present in the ALCAPA patients.

**Table 3:** Comparison of the echocardiographic parameters of diastolic function in the study population at last follow-up and the control group.

Parameter	ALCAPA (n = 18)	Control group (n = 18)	p *
Mitral E [m/s]	0.97 ± 0.19	0.99 ± 0.19	0.83
DTE [ms]	165 ± 36	163 ± 30	0.87
**Mitral A [m/s]**	**0.61 ± 0.14**	**0.51 ± 0.13**	**0.03**
**E‘ [cm/s]**	**12 ± 2**	**17 ± 2**	**<0.00001**
**E/E‘**	**8.1 ± 2.6**	**5.8 ± 1.3**	**<0.001**
**Pulm Vein S [m/s]**	**0.39 ± 0.12**	**0.51 ± 0.11**	**<0.01**
**Pulm Vein D [m/s]**	**0.71 ± 0.11**	**0.60 ± 0.11**	**0.01**
**Pulm Vein S/D**	**0.56 ± 0.18**	**0.86 ± 0.23**	**<0.001**
**Pulm Vein A [m/s]**	**0.44 ± 0.14**	**0.30 ± 0.04**	**<0.01**

Mitral E / Mitral A–pulsed wave Doppler early / atrial mitral velocity, DTE–deceleration time of early mitral velocity, E’–spectral tissue Doppler early diastolic velocity (mean of the basal segment of the lateral wall and interventricular septum), Pulm Vein S / D / A–pulmonary veins maximal systolic / diastolic / atrial velocity

* Student’s t test for independent samples

The most striking abnormalities were found in the peak LS measurements (Table 4). The mean LV_GLS was -15.8 ± 3.3% in the ALCAPA group and -21.9 ± 1.7% in the control group (p<0.00001). The mean LS in the ALCAPA patients in the RCA region was -19.0 ± 4.4%, and in the LCA region it was -13.8 ± 7.3% (p<0.00001). There were significant differences in the strain parameters between the ALCAPA and control group not only in the LCA region, but also in the RCA region. The only two segments with comparable LS values between the groups were the basal segments of the inferior and septal wall, both in the RCA region. The worst peak LS was present in the basal lateral LV segments (-8.9 ± 8.9%), however abnormal LS (defined as ≥ -15.9%) was found in the whole LV anterior and lateral wall, basal and middle segments of the posterior wall and the apex. Each patient in the ALCAPA group showed at least one LV segment with abnormal LS value. Also the LS values obtained from the RV and both atria differed significantly in comparison to the healthy controls. Radial and circumferential LV strain, however, was comparable between the ALCAPA and the control group.

**Table 4:** Comparison of the 2-dimensional echocardiographic longitudinal speckle tracking strain parameters in the study population at last follow-up and the control group.

LV region / segment [%]		ALCAPA (n = 18)	Control group (n = 18)	p *
Right coronary artery (RCA) region	Inferior basal LS	-19.8 ± 3.9	-21.8 ± 3.1	0.09
	Inferior mid LS	-20.7 ± 3.5	-23.3 ± 3.4	0.03
	Inferior apical LS	-17.3 ± 7.0	-24.7 ± 3.9	<0.001
	Septal basal LS	-18.2 ± 2.2	-18.2 ± 2.7	1.00
	Septal mid LS	-19.3 ± 3.4	-21.3 ± 2.1	0.03
	Mean RCA region	-19.0 ± 4.4	21.9 ± 3.8	<0.00001
Left coronary artery (LCA) region	Ant_septal basal LS	-16.5 ± 4.4	-20.1 ± 3.2	<0.01
	Ant_septal mid LS	-16.6 ± 5.6	-22.4 ± 3.3	<0.001
	Anterior basal LS	-11.9 ± 6.5	-21.5 ± 3.5	<0.00001
	Anterior mid LS	-14.1 ± 4.7	-23.8 ± 4.2	<0.00001
	Anterior apical LS	-15.8 ± 8.2	-23.9 ± 5.1	<0.01
	Lateral basal LS	- 8.9 ± 8.9	-22.0± 3.4	<0.00001
	Lateral mid LS	-11.6 ± 7.6	-22.9 ± 3.8	<0.00001
	Lateral apical LS	-13.9 ± 6.5	-22.2 ± 4.2	<0.0001
	Posterior basal LS	-12.8 ± 5.9	-22.2 ± 3.5	<0.00001
	Posterior mid LS	-14.5 ± 4.0	-21.8 ± 2.4	<0.001
	Septal apical LS	-16.0 ± 9.2	-23.2 ± 4.1	<0.01
	Apex LS	-12.8 ± 11.2	-23.4 ± 3.9	*<0*.*0001*
	Mean LCA region	-13.8 ± 7.3	-22.5 ± 3.8	<0.00001
GLS_LV [%]		-15.8 ± 3.3	-21.9 ± 1.7	<0.00001
GLS_APLAX [%]		-15.2 ± 3.0	-21.7 ± 1.9	<0.001
GLS_ 4Ch [%]		-14.1 ± 4.4	-21.5 ± 2.4	<0.00001
GLS_2Ch [%]		-16.9 ± 3.3	-22.5 ± 2.6	<0.00001
Radial LV strain [%]		43.1 ± 18.5	41.6 ± 11.1	*0*.*71*
Circumferential strain [%]		16.3 ± 4.2	17.1 ± 2.8	0.51
Other cardiac chambers:				
RV_LS [%]		-20.6 ± 3.9	-24.9 ± 4.6	<0.01
LA_LS [%]		27.7 ± 4.3	41.0 ± 11.5	<0.0001
RA_LS [%]		26.8 ± 7.4	44.0 ± 7.9	<0.00001

LV–left ventricle, LS–peak longitudinal strain, ant–anterior, GLS–LV global LS, GLS_APLAX–GLS in apical long axis view, GLS_4Ch–GLS in apical 4 chamber view, GLS_2Ch–GLS in apical 2 chamber view, RV–right ventricle, LA–left atrium, RA–right atrium

* Student’s t test for independent samples or Mann Whitney U test (italics)

Longitudinal LV strain could not be calculated only in 8 out of the 612 segments studied (99% accessibility). Right ventricular and RA strain were accessible in 97% of patients, while LA strain in 100%. The least reliable strain parameters were the LV circumferential and radial strain, which could be adequately tracked in 86% of patients.

The comparison between 4 patients after Takeuchi repair and the 14 patients after direct coronary translocation did not reveal any major differences in LVEF or other precise parameters describing cardiac function, such as the LV, RV or atrial strain (Table 5). However, patients after the Takeuchi repair were significantly older, showed higher maximal pulmonary artery velocity and one of them required reoperation due to baffle leak.

**Table 5:** Comparison of the major echocardiographic parameters between patients with direct reimplantation of the anomalous left coronary artery and patients who underwent the Takeuchi repair at last follow-up.

Parameter	Coronary artery translocation (n = 14)	Takeuchi repair (n = 4)	p *
**Age [years]**	**14.4 ± 8.1**	**24.2 ± 4.7**	**0.04**
BSA [m^2^]	1.41 ± 0.18	1.65 ± 0.07	0.34
LVEF	56.0 ± 4.8	51.7 ± 8.9	0.21
LVEDvol [ml]	81.1 ± 35.3	103.2 ± 41.1	0.30
GLS_LV [%]	-16.0 ± 3.7	-15.2 ± 1.5	0.68
RV_LS [%]	-20.4 ± 3.8	-21.5 ± 4.8	0.62
LA_LS [%]	28.8 ± 4.2	27.2 ± 5.3	0.83
RA_LS [%]	26.7 ± 7.7	27.3 ± 7.1	0.90
**Vmax Pulm [m/s]**	**1.41 ± 0.68**	**2.54 ± 0.88**	***<0*.*05***

For abbreviations see Table 4; Vmax Pulm–maximal pulmonary artery systolic velocity

* Student’s t test for independent samples or Mann Whitney U test (italics)

## Discussion

Our study is the one with the longest follow-up concerning the ALCAPA patients operated in their early childhood, with the use of STE. The vast majority of patients remained in good clinical condition, normalized their LVEF, diastolic cardiac dimensions, and diminished the severity of mitral regurgitation without mitral valve surgery [15], similarly to what others have reported [2,13]. Despite no significant stenosis of the proximal LCA on color Doppler, detailed echocardiographic analysis revealed extensive abnormalities in comparison to the age, sex and BSA-matched control group. The hypothesis concerning improvement of LVEF in ALCAPA patients after surgery with persistence of subtle abnormalities due to infarcted, fibrosed cardiac myocytes has been studied before, but the present study is more comprehensive than the previous ones [9, 11, 16, 23]. We revealed a significant discrepancy between the commonly used robust parameter of LV systolic function such as LVEF and the more sensitive ones, like tissue Doppler and especially LS. Apart from abnormal longitudinal function of all cardiac chambers, we confirmed the persistence of impaired LV diastolic function and the presence intraventricular dyssynchrony. Additionally, LS abnormalities were not confined to the myocardium perfused by the LCA, but were also present in the RCA region. All these findings underline the extensiveness of myocardial damage detectable by detailed echocardiography.

The ALCAPA syndrome is a unique clinical model to study the influence of chronic hypoperfusion and subsequent reperfusion on the LV function, without the confounding effects of comorbidities, like hypertension, tobacco smoking, diabetes or dyslipidemia. One patient of whole 24-cases group under observation in our university hospital died suddenly 21 years after surgery—no abnormalities were present in her clinical status or standard echocardiographic examination at the last follow-up before death [15]. This case suggested that in this patient group standard follow-up parameters fail to identify patients at risk and the long-term postoperative prognosis is uncertain. In further observation of our ALCAPA group, two other asymptomatic patients with normal (57%) or slightly impaired (51%) LVEF, but abnormal global LS (-15% and -14,8%, respectively) presented malicious ventricular arrhythmias (nonsustained and sustained ventricular tachycardia), which were detected by the 30-day wearable ambulatory ECG monitoring. Ischemia may cause irreversible injury with an arrhythmogenic potential of the post-ischemic tissue [24].

In agreement with previous studies [2,13], the LVEF in our ALCAPA population recovered after surgical repair and remained within normal range in the long-term follow-up. More sensitive parameters, based on STE, showed impaired LS of all cardiac chambers, which was probably a manifestation of chronically hypoperfused subendocardial longitudinally-oriented fibres most susceptible to ischemia [25]. Potential causes of persistent LS abnormalities could be myocardial ischemia due to preoperative coronary steal phenomenon or perioperative myocardial injury. Also the tissue Doppler parameters of longitudinal LV and RV function (S’LW, S’IVS, S’RV, TAPSE) in our study population showed impairment in comparison to the control group. Interestingly, RV function recovered less in the long-term follow-up in those patients who showed more severely impaired LV function before operation.

Neonates with ALCAPA syndrome develop some degree of myocardial necrosis soon after the birth due to changes in cardiopulmonary circulation and the absence of adequate collateralization, resulting in severe ischemia and LV dysfunction. Successful revascularization initiates the process of reverse LV remodeling, but despite normalization of LV global function after surgery, residual coronary lesions or fibrosis may exist. Finally the myocardium in ALCAPA patients can be chronically hypoperfused. Despite the LV function recovery in the conventional echocardiographic evaluation after restoration of a dual coronary artery system, the STE analysis revealed important functional abnormalities within all cardiac chambers. The STE technique measures regional ventricular deformation, is independent from ventricular geometry, and can identify subclinical dysfunction at earlier stages than conventional echocardiography [26–28].

Although data on strain analysis in the pediatric and young adult population is limited, there are some reports on its usefulness in the ALCAPA population. Mertens et al. reported on the utility of serial strain measurements in an infant after ALCAPA repair, in who after the surgery the recovery of longitudinal function was delayed when compared with radial function [29]. Similarly, Di Salvo et al. [30] showed persistent abnormal longitudinal myocardial strain in 30 patients assessed at least 12 months after ALCAPA repair. In contrast, Cabrera et al. [9] showed decreased both: global longitudinal and circumferential strain in 11 out of 14 ALCAPA patients (79%) examined at (median) 6 years after surgical repair. In our study, only the LS parameters remained impaired, while the radial and circumferential LV strain values, which reflect more the function of the oblique and circumferential fibres of the LV, did not differ in comparison to the control group. However, similarly to previous studies, we confirmed the vulnerability of subendocardial longitudinal fibers to ischemic injury and the excellent sensitivity of LS to reveal it.

Abnormal LS in our study patients was predominantly found in the LCA region, similarly to other ALCAPA populations studied. In the study by Secinaro et al. [31] performed with the use of the cardiac magnetic resonance imaging in 6 patients aged 6–21 years, the abnormalities were localized in the LV antero-lateral wall. In our study, the impaired LS was located predominantly in the LV postero-lateral wall, however in contrast to other studies [9,11, 30] regional abnormalities were present also in the RCA region. Additionally, some authors [16, 31] observed sparing of the apical segments after ALCAPA repair, while patients in our study showed akinetic apical segments and severely abnormal LS in this region. The LS parameters in the ALCAPA population were comparable with the control group only in the LV basal inferior and septal segments.

Mean LVEDd normalized at follow-up, which can result from long-term effects of normalized coronary blood flow. At follow-up LVESV was greater in the ALCAPA group, while LVEDV did not differ between the ALCAPA and the control group. This can be related to areas of potentially irreversible LV damage in the LCA territory. Relative hypertrophy of the posterior wall could also be due to the remodeling process in the ALCAPA group, with segments supplied by RCA potentially free of areas of irreversible damage as opposed to the segments in the LCA territory in which hypertrophy was less pronounced.

Our study reports also on the impaired LV diastolic function in the ALCAPA patients, which can be due to increased LV stiffness. Decreased LA strain and tissue E’ values, increased E/E’ ratio and mitral A velocity, as well as all parameters of the pulmonary vein flow reflected abnormal diastolic function, in comparison to the control group. This is in agreement with previous studies [29], however, our study is the first assessing LA strain and pulmonary vein flow in the ALCAPA population. Impaired RA and RV strain, not studied previously, complement to the LV functional abnormalities. They are probably a result of pericardiotomy in anamnesis, secondary to diastolic LV dysfunction or the coronary steal phenomenon present preoperatively also in the RCA area.

Mitral valve regurgitation due to valvular annular dilation or papillary muscle ischemia was present in the majority of our patients before operation, but after restoration of proper coronary circulation, even without valvular surgery, the insufficiency became hemodynamically insignificant. Only fibroelastosis of the papillary muscles persisted in the long-term follow-up.

Four patients from our study group underwent Takeuchi repair. It was reported that patients after Takeuchi repair show higher incidence of pulmonary stenosis [32], which was also the case in our series. Maximal velocity through the pulmonary valve was significantly higher in patients after Takeuchi repair, and in 3 out of 4 patients it was higher than 2 m/s. One patient with pulmonary stenosis had baffle leak which required reoperation. None of the patients had baffle stenosis. Patients after Takeuchi repair did not differ with regard to other parameters related to ventricular or atrial function in comparison to the group with direct coronary translocation.

### Limitations of the study

The follow-up duration was not uniformly scheduled for all patients, thus we could not determine the exact time to the LV recovery after surgery. The observer could not be blinded while obtaining the STE data. In addition, there are some technical limitations of image acquisition and strain imaging in small children, with potential measurement variability. Control coronary angiography was not performed in the follow-up and the patency within the proximal coronary arteries was done predominantly on the basis of color Doppler echocardiography, however 13 patients underwent cardiac magnetic resonance and 9 cardiac computed tomography imaging–both examinations ruled out significant stenoses. The sample size of the study was relatively small, but regarding low incidence of the ALCAPA syndrome in the general population, this is a rather typical cohort size, especially considering the long observation time.

## Conclusions

In conclusion, in spite of good clinical condition and normalization of the standard echocardiographic parameters in the long-term observation of the ALCAPA patients after the surgical repair, more precise techniques revealed persistent abnormalities in cardiac function. Impaired diastolic, and LV longitudinal systolic function and subtle regional LV abnormalities, persisted many years after surgical repair, even in the presence of normal LVEF. The LV deformation abnormalities were predominantly located in the LCA region, but the RCA region was also affected. The results from our study underline the necessity of constant survey of the ALCAPA patients after surgical repair. We recommend extensive echocardiographic examination with tissue Doppler and strain evaluation in addition to standard parameters in these patients. Further studies are needed to assess clinical significance and prognostic value of the persistent subtle echocardiographic abnormalities.

## Supporting information

S1 Table Patients’ demographics and clinical data at the time of diagnosis. (ZIP)

## Abbreviations

ALCAPA: anomalous origin of the left coronary artery from the pulmonary artery
E and A: early and atrial wave velocity of the mitral inflow assessed by pulsed-wave Doppler
E: peak early diastolic velocity by spectral tissue Doppler
LCA: left coronary artery
LV: left ventricle
LVEDd / LVESd: left ventricular end-diastolic / end-systolic diameter
LVEF: left ventricular ejection fraction
LV_GLS: left ventricular global longitudinal strain
LS: longitudinal strain
PSD: peak strain dyspersion
RCA: right coronary artery
RV: right ventricle
STE: speckle tracking echocardiography
S’LW / S’IVS / S’RV: peak systolic velocities in the basal segments of lateral left ventricular wall / interventricular septum / lateral right ventricular wall
TAPSE: tricuspid annular plane systolic excursion
2D: two-dimensional
