# Supplementary material for: Myocardial function in patients with anomalous left coronary artery from the pulmonary artery syndrome: A long-term speckle tracking echocardiographic study
Source: PLoS One. 2019 Oct 15;14(10):e0223227. doi: 10.1371/journal.pone.0223227 (PMC6793868; doi:10.1371/journal.pone.0223227)
Supplement: S1 Table — (ZIP) [file pone.0223227.s001.zip › S1 Table.docx..docx]

**Supplementary Table 1.** **Patients’ demographics and clinical data at the time of diagnosis (n=18)**

| Parameter | mean (± standard deviation) / median (range)  or number of patients (percentage) |
| --- | --- |
| Age at diagnosis [months] | 25 ± 46 / 6 (3 – 156) |
| Reason for hospital admittance:  enlarged heart silhouette on the chest X-ray  pneumonia and heart failure  systolic murmur  dyspnoea/fatigue  cardiogenic shock  arrhythmia  suspicion of myocarditis  failure to thrive  control echocardiography after VSD closure | 6 (33%)  3 (16%)  2 (8%)  2 (8%)  1 (4%)  1 (4%)  1 (4%)  1 (4%)  1 (4%) |
| General condition at recognition:  good  average  severe | 8 (44%)  3 (17%)  7 (39%) |
| Additional congenital heart defects:  VSD  ASD II  PDA  mild aortic stenosis | 1 (4%)  1 (4%)  1 (4%)  1 (4%) |
| Echocardiographic parameters before cardiac surgery: | |
| LVEF [%] | 33 ± 17 / 27 (14 – 69) |
| Mitral insufficiency (n=17):  mild  moderate  severe | 3 (18%)  10 (56%)  4 (24%) |
| Fibroelastosis of the papillary muscles (n=14) | 14 (100%) |
| Age at cardiac surgery [months] | 28 ± 49 / 7 (3 – 167) |
| Surgical procedure:  direct aortic reimplantation of the ALCAPA Takeuchi repair | 14 (78%)  4 (22%) |
| Major complications after surgery | 12 (67%) |
| LVEF 1 year after cardiac surgery | 65 ± 11 / 66 (30 – 83) |

ALCAPA – anomalous left coronary artery from the pulmonary artery, LVEF – left ventricular ejection fraction determined by the Teicholz method, VSD – ventricular septal defect, ASD II – atrial septal defect second type, PDA – patent ductus arteriosus
